# In Vivo Investigation into Effectiveness of Fe_3_O_4_/PLLA Nanofibers for Bone Tissue Engineering Applications

**DOI:** 10.3390/polym10070804

**Published:** 2018-07-22

**Authors:** Wei-Yi Lai, Sheng-Wei Feng, Ya-Hui Chan, Wei-Jen Chang, Hsin-Ta Wang, Haw-Ming Huang

**Affiliations:** 1 School of Organic and Polymeric, National Taipei University of Technology, Taipei 10608, Taiwan; jefflai28@hotmail.com; 2 School of Oral Hygiene, College of Oral Medicine, Taipei Medical University, Taipei 11031, Taiwan; b8702070@tmu.edu.tw; 3 School of Dentistry, College of Oral Medicine, Taipei Medical University, Taipei 11031, Taiwan; babypg@hotmail.com (Y.-H.C.); m8404006@tmu.edu.tw (W.-J.C.); 4 Dental Department, Taipei Medical University Shuang-Ho Hospital, New Taipei City 23561, Taiwan; 5 Graduate Institute of Biomedical Optomechatronics, College of Biomedical Engineering, Taipei 11031, Taiwan

**Keywords:** Fe_3_O_4_, poly-l-lactide, electrospinning, osteogensis

## Abstract

Fe_3_O_4_ nanoparticles were loaded into poly-l-lactide (PLLA) with concentrations of 2% and 5%, respectively, using an electrospinning method. In vivo animal experiments were then performed to evaluate the potential of the Fe_3_O_4_/PLLA nanofibrous material for bone tissue engineering applications. Bony defects with a diameter of 4 mm were prepared in rabbit tibias. Fe_3_O_4_/PLLA nanofibers were grafted into the drilled defects and histological examination and computed tomography (CT) image detection were performed after an eight-week healing period. The histological results showed that the artificial bony defects grafted with Fe_3_O_4_/PLLA nanofibers exhibited a visibly higher bone healing activity than those grafted with neat PLLA. In addition, the quantitative results from CT images revealed that the bony defects grafted with 2% and 5% Fe_3_O_4_/PLLA nanofibers, respectively, showed 1.9- and 2.3-fold increases in bone volume compared to the control blank sample. Overall, the results suggest that the Fe_3_O_4_/PLLA nanofibers fabricated in this study may serve as a useful biomaterial for future bone tissue engineering applications.

## 1. Introduction

Bone defects are traditionally treated using autogenous bone grafts. However, while bone grafts have the advantages of a low infection risk and a high fracture healing rate [1], it is difficult to obtain a sufficient bone mass for large bone defects due to individual patient factors [1,2]. Thus, an increasing number of synthetic bone filling materials have been developed over the years [1,3,4]. These materials typically have both good biocompatibility and superior physical properties [1]. As a result, they have been widely used in the treatment of small-scale bone defects and alveolar bone filling after tooth extraction [2,3,5,6,7]. Many studies have shown that these artificial materials promote osteogenetic differentiation [1] and enhance the control growth factor expression [1] of mesenchymal stem cells [8,9]. As a result, they have attracted significant attention in the literature.

Natural polymers—such as collagen, gelatin, silk, and chitosan—are efficacious materials for bone tissue regeneration [1,3]. Besides natural polymers, synthetic materials, such as polylactic acid (PLA), also have a promising potential for bone regeneration applications. For example, previous studies have shown that rat bone marrow cells cultured on PLA exhibit significant type I collagen expression and calcium mineralization after four weeks of healing [3,5,10]. Badami et al. (2006) similarly reported that osteoblastic cells cultured with poly-l-lactide (PLLA) nanofibers showed excellent osteogenetic activity [11].

The performance of composite materials in tissue engineering applications is dependent to a large extent on the physical properties and surface characteristics of the particular material concerned. For example, the degradation of synthetic polymers depends on their crystallinity, molecular weight, and mass in the biological tissue [4,12,13]. However, synthetic materials with an appropriate porosity and structure are extremely helpful in increasing cell proliferation and differentiation [4,5,12]. Among the various synthetic materials available, PLLA has high hydrophobicity and is thus particularly conducive to cell attachment due to its potential to provide good adhesion and growth environment for bone cells [4,12,13].

The major function of bone grafts is to provide a suitable environment to induce bone cell growth in bone defects. Extracellular matrix materials provide an ideal environment for osteoblastic proliferation and regeneration and new blood vessel formation. Consequently, many nanofibers have been manufactured for use as bone grafts in recent years [4,8,9,12,14,15]. Many studies have shown that nanofibrous scaffolds stimulate mineralization, bone regeneration, and tissue interaction in the bone healing process [16,17,18,19]. For the methodology, electrospinning technique was the most common method for fabricating polymer nanofibers [12,20]. Martins et al. (2010) manufactured composite scaffolds containing chitosan using an electrospinning technique and found that the resulting fibers can serve not only as a scaffold, but also as a growth factor release system [20]. Bone scaffolds produced using the electrospinning technique also have a positive effect on the attachment, proliferation, and differentiation of osteoblastic cells [20,21,22]. Similarly, PLLA nanofibers made by electrospinning have an excellent three-dimensional structure and porosity, and are extremely effective in promoting bone cell attachment, proliferation, and regeneration [5].

Various nano-magnetic particles have been developed for biological applications in recent years [23,24,25]. Among these materials, nano-magnetite (Fe_3_O_4_) has particularly good biocompatibility and functionality for medical applications. In 2006, Kim et al. injected nano-Fe_3_O_4_ particles into mice and found that the particles could be detected in the brain and had no toxic effects [23]. Moreover, several authors [24,25] showed that the unique magnetic properties of nano-magnetite particles render then an ideal material for a wide range of medical applications, including drug delivery, magnetic resonance imaging (MRI), magnetic thermotherapy, cell separation, protein separation, and DNA detection. Nano-magnetite particles have also been reported to increase the osteoinductive effect [26,27,28]. Notably, several studies have shown that the integration of nano-magnetite particles with polymer fibers not only solves the problem of agglomeration, but also improves their stability in biological applications [24,25].

In 2014, Wang et al. developed a novel composite material consisting of magnetic nanoparticles loaded into PLLA by means of electrospinning [29]. Fibroblast cells cultured on the magnetized Fe_3_O_4_/PLLA nano-membranes exhibited a good spreading and attachment behavior. Thus, the authors suggested that nano-magnetite/PLLA fibrous material has a good potential for tissue engineering applications [29]. However, in vivo tests of the fibrous material were not performed. Accordingly, the present study performs an in vivo investigation into the effectiveness of nano-magnetite Fe_3_O_4_/PLLA fibers for bone tissue engineering applications using an animal model.

## 2. Materials and Methods

### 2.1. Electrospun Nano-Fe_3_O_4_/PLLA Composites

Fe_3_O_4_ nanoparticles (99.9% purity, 50 nm in diameter) were purchased from Long Ton Inc., Taipei, Taiwan. PLLA particles (molecular weight of 100 kDa, Wei Mon Industry Co., Taipei, Taiwan). Fe_3_O_4_/PLLA nano-composites were prepared according to the method described in a previous report [30]. Briefly. before mixing the Fe_3_O_4_ nanoparticles and PLLA powder, the two materials were dried at 80 °C for 24 h. A extruder (Twin-screw extruder, Plastics Industry Development Center, Taichung City, Taiwan) was then used to mix the two materials at a temperature of 150 °C. After cooling the extruded composite strands to 25 °C, small granules were prepared with a pelletizer. The non-cytotoxic property of this nanoparticles/PLLA composite has been proven in our previous works [31,32] and a cell culture experiment in Figure S1.

In this study, three nano-Fe_3_O_4_/PLLA composites were prepared with Fe_3_O_4_ to PLLA mixing ratios of 0%, 2%, and 5% (*w*/*w*). The mixtures were dissolved in a dichloromethane/dimethylformamide (DCM/DMF) solution (4:1 *v*/*v*) with a concentration of 10% (*v*/*v*). The dissolved materials were then poured into 15-mL syringes fitted with stainless steel needles with an internal diameter of 0.8 mm. In performing the electrospinning process, the needles were connected to a high voltage power supply (20 kV), while the collector plate was connected to a grounding circuit. For each composite material, the collecting plate distance was set as 15 cm and the flow rate was set equal to 1.0 mL/h. The ultrastructures of the electrospun membranes were examined using a scanning electron microscope (S-2400, Hitachi, Hitachi, Ltd., Tokyo, Japan) coupled with energy dispersive X-ray spectroscopy (EDX) (EMAX ENERGY, Horiba, Kyoto Ltd., Kyoto, Japan). The thickness of the nanofibers was examined by measuring 30 individual nanofibers in random SEM images using image analysis software (Image-Pro Plus, Media Cybernetics, Silver Spring, MD, USA).

### 2.2. Animal Experiments

**Figure 1:**
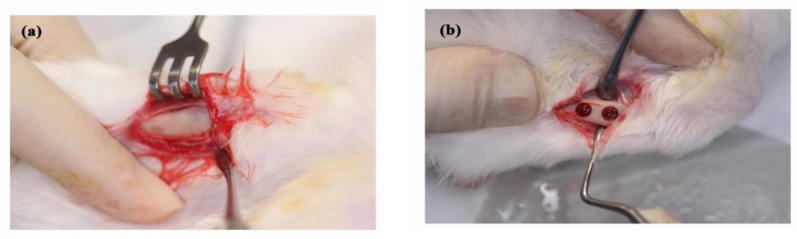

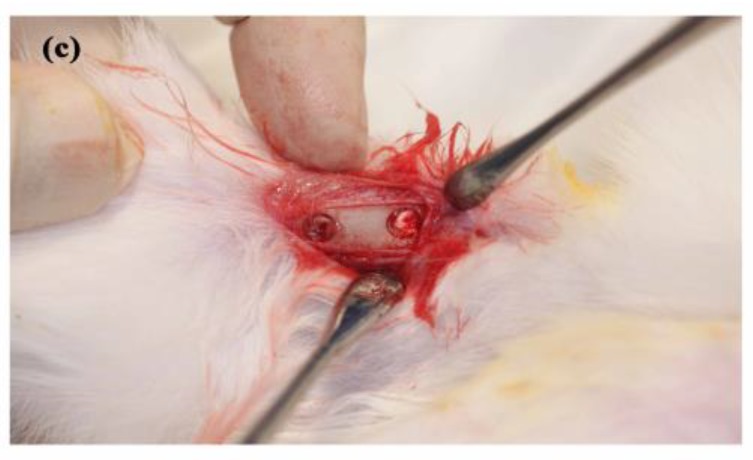
Procedure of animal experiment. (**a**) The tibia of the rabbit was scraped, (**b**) two implantation holes with a diameter of 4 mm were prepared at each tibia of the rabbit, (**c**) neat PLLA was grafted into one implantation site and Fe_3_O_4_/PLLA nanofibers were grafted into the other.

Twelve New Zealand white rabbits (2.5–3.0 kg in weight) were used as test subjects. The study protocol and surgical procedure were approved by “The Care and Use of Laboratory Animals of Taipei Medical University” (LAC-2014-0245). Prior to the experiments, the rabbits were fed solid food and maintained in bracket cages at a temperature of 20 °C and a humidity of 50%. The surgical procedures were performed under sterile conditions. General anesthesia was performed via the intramuscular injection of tiletamine-zolazepam (Zoletil 50, Virbac, Carros CEDEX, France) at a dosage rate of 15 mg/kg. The operation area was shaved and local anesthesia was achieved by injecting epinephrine subcutaneously at the surgical site. The tibia surface was exposed after skin incision and muscle dissection (Figure 1a). Implantation holes with a diameter of 4 mm were prepared using an electronic driller. The holes were drilled to a depth of 5 mm under saline cooling conditions. Two implant holes were prepared in each side of tibia (Figure 1b). The two holes on the right tibia were grafted with 0% (neat PLLA) and 5% nano-Fe_3_O_4_ /PLLA nanofibers, respectively, while the two holes on the left tibia were used for ungrafted control and 2% nano-Fe_3_O_4_ /PLLA nanofiber implantation, respectively. For each hole, 10 mg of sample was grafted (Figure 1c). After grafting, the muscle and skin were closed with absorbable sutures (Vicryl^®^ 4.0, Ethicon, Somerville, MA, USA) and postoperative antibiotics and analgesics were administered intramuscularly for three days. The bone healing condition was then observed after eight weeks. At observation time, the rabbits were sacrificed under general anesthesia by Zoletil 50 (50 mg/mL) at a dosage of 15 mg/kg following CO_2_ gas asphyxiation. Bone tissue was collected from the surgical areas and fixed in a 10% formaldehyde solution at pH 7.0 for further analysis. In this study, none of the rabbits showed moribund conditions during the experimental period.

### 2.3. Micro-CT Measurements

To observe the bone healing condition, the collected samples were scanned in a micro-computed tomography (micro-CT) scanner (SkyScan 1076, Bruker, Kontich, Belgium) using an energy level of 55 kV and a pixel resolution of 18 µm. Three-dimensional images were reconstructed using CTan software (Bruker). For each sample, the bone volume was identified in accordance with selected threshold values (grayscale density between 60 and 140). Furthermore, as in previous studies, the bone volume was quantitated by calculating the ratio of the bone volume (BV) to the total tissue volume (TV) in the implantation holes [33,34,35].

### 2.4. Histological Analysis

For each sample, the bone growth condition was evaluated by means of histological analyses. Briefly, the bone samples were decalcified in decalcifier (Thermo Scientific™ Shandon™ TBD-1™, Cheshire, UK) for four weeks and then dehydrated in an ascending alcohol gradient (60–100%). The samples were embedded in paraffin and cut into sections with a thickness of 5 µm. Finally, the specimens were stained with hematoxylin and eosin and histological images were acquired using a microscope slide scanner (OPTIKA, Ponteranica, Italia). A commercialized image software (Image-Pro Plus 4.5, Media Cybernetics, Silver Spring, MD, USA) were performed to quantify the new bone ratio of the images.

### 2.5. Statistical Analysis

The mean values and standard deviations of the BV/TV values were obtained. Differences between the various samples and the blank control group were investigated by means of one-way analysis of variance (ANOVA) tests (SPSS Inc., Chicago, IL, USA) with LSD post hoc. In all of the tests, statistical significance was defined as a *p* value less than 0.05.

## 3. Results

**Figure 2:**
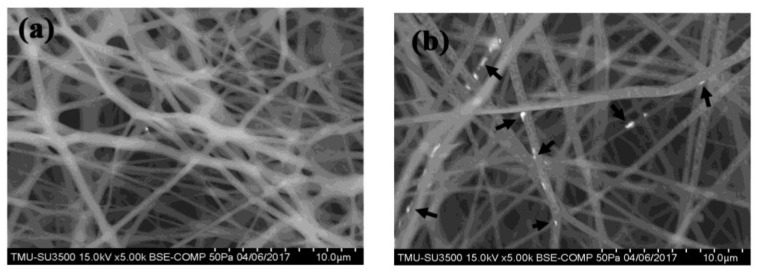
SEM microstructures of electrospun PLLA nanofibers incorporating Fe_3_O_4_ nanoparticles. (**a**) Neat PLLA nanofibers; and (**b**) Fe_3_O_4_ nanoparticles (black arrows) integrated within PLLA nanofibers.

**Figure 3:**
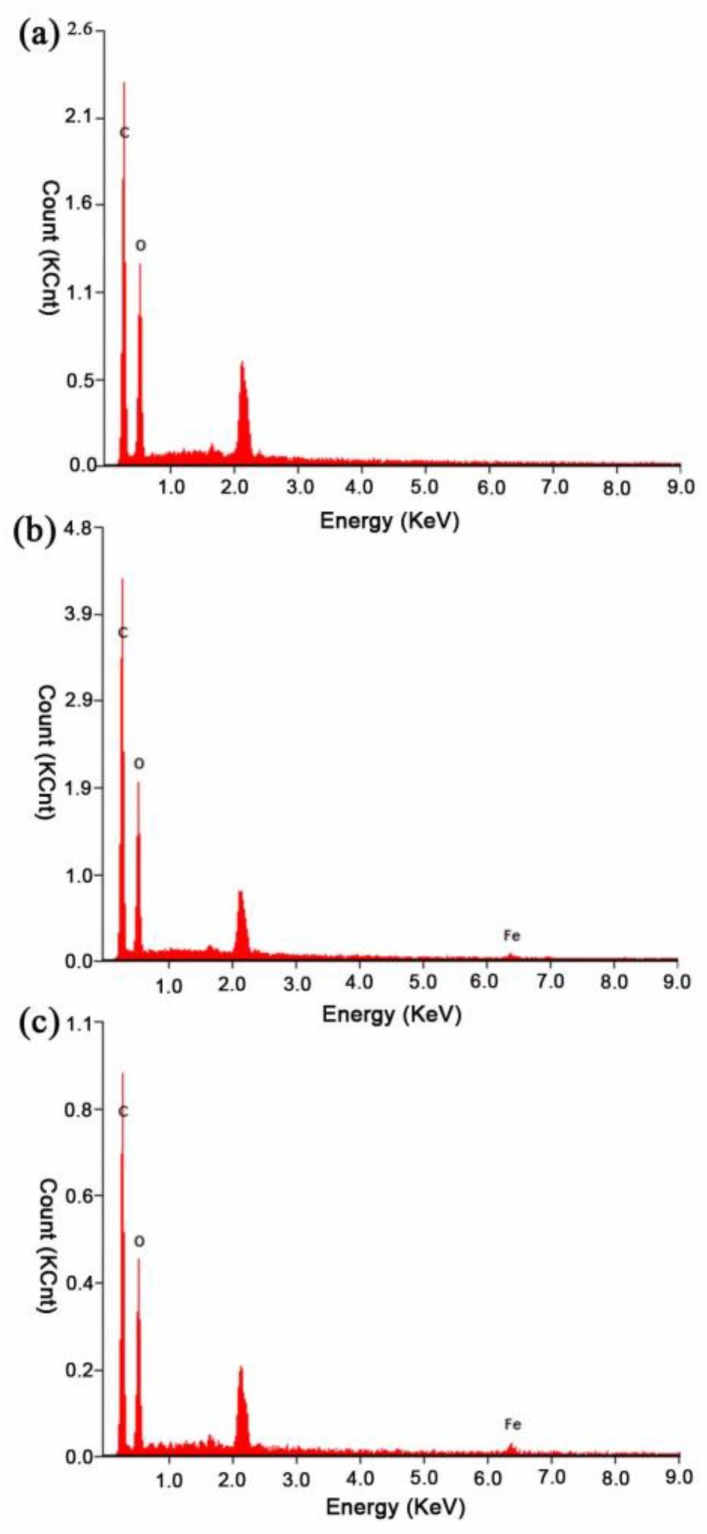
Energy dispersive X-ray spectroscopy results for surface compositions of: (**a**) neat PLLA nanofibers; (**b**) 2% Fe_3_O_4_/PLLA nanofibers; and (**c**) 5% Fe_3_O_4_/PLLA nanofibers. The *y*-axis shows the count number of X-rays received by the detector (with a unit of 1000 count, KCnt) and the *x*-axis shows the energy level of the peaks (with a unit of kiloelectron volts, KeV).

Figure 2 shows the SEM microstructures of the neat PLLA nanofibers (Figure 2a) and the Fe_3_O_4_/PLLA nanofibrous network (Figure 2b). The thickness of the fibers ranged from 300 to 1600 nm, while the diameters of the neat PLLA nanofibers vary in the range of 780 ± 358 nm, and are thus significantly smaller than those of the fibers containing 2% Fe_3_O_4_ nanoparticles (1001 ± 188 nm) and 5% Fe_3_O_4_ nanoparticles (945 ± 212 nm), respectively. The EDX analysis results show that the Fe_3_O_4_/PLLA nanofibers with 2% and 5% Fe_3_O_4_ addition have Fe contents of 2.27 ± 0.56% and 5.06 ± 1.33%, respectively (Figure 3).

**Figure 4:**
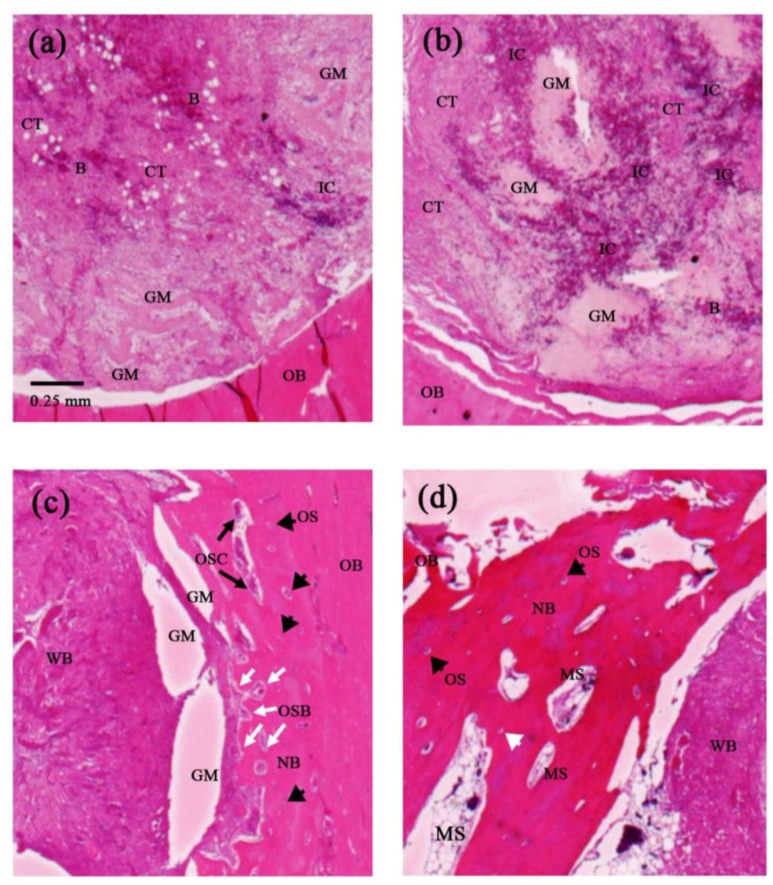
Histological images of samples grafted with 5% Fe_3_O_4_/PLLA nanofibers after: (**a**) one; (**b**) two; (**c**) four; and (**d**) eight weeks. B: blood, CT: connective tissue. GM: grafted material, IC: inflammatory cell, MS: marrow space, NB: new bone, OB: old bone, OS: osteocyte, OSB: osteoblast, OSC: osteoclast, WB: woven bone. Scale bar: 250 μm.

Figure 4 presents histological images of the bone defect grafted with 5% Fe_3_O_4_/PLLA nanofibers after healing periods of one, two, four, and eight weeks, respectively. After one week, blood clots and inflammatory cells are observed in the central area of the grafting site (Figure 4a). Moreover, loose fibrous connective tissue is also found. After two weeks, a severe inflammation response occurs and dense fibrous connective tissue is noted within the grafting area (Figure 4b). Four weeks after implantation, marked woven bone and new mature bone formation are observed in the central and marginal areas of the grafting site, respectively (Figure 4c). Moreover, osteocytes are seen within the substance of the newly-formed bone, while activated osteoblast and osteoclast are found at the interface between the woven bone and the newly-formed bone (Figure 4c). Finally, after eight weeks, a more extensive growth of new mature bone is observed in the marginal area of the grafting site and bone marrow cavity formation is noted in the newly-formed bone for the first time (Figure 4d).

**Figure 5:**
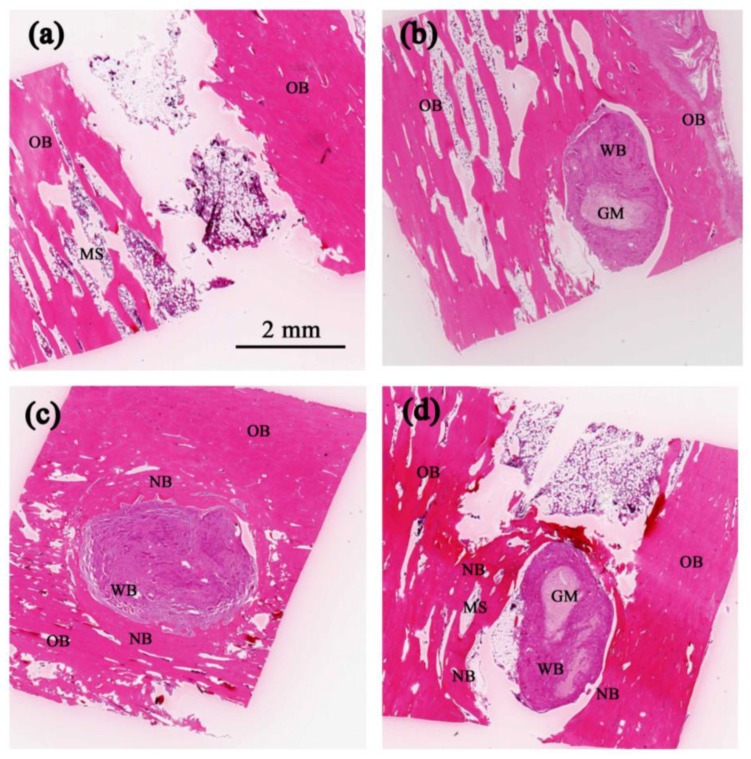
Histological images of samples grafted with: (**a**) blank; (**b**) neat PLLA; (**c**) 2% Fe_3_O_4_/PLLA; and (**d**) 5% Fe_3_O_4_/PLLA nanofibers after eight weeks. Scale bar: 2 mm.

Figure 5 presents histological images of the blank, neat PLLA, 2% Fe_3_O_4_/PLLA, and 5% Fe_3_O_4_/PLLA nanofiber samples after a healing period of eight weeks. An observation of Figure 5a shows that the grafting-free bone defect contains only loose fibrous connective tissue. However, the defect grafted with neat PLLA nanofibers contains abundant woven bone (Figure 5b). Visible bone formation is observed in the marginal area of the defect grafted with 2% Fe_3_O_4_/PLLA nanofibers (Figure 5c). The defect grafted with 5% Fe_3_O_4_/PLLA nanofibers also contains visible bone formation. Notably, the new bone/woven bone ratio is higher than that for the 2% Fe_3_O_4_/PLLA nanofiber sample and bone marrow cavities are observed within the newly-formed bone (Figure 5d). Quantification of the new bone formation was listed in Table 1. The ratio of new formed bone reached 43% when the amount of added Fe_3_O_4_ nanoparticles was larger than 2%.

**Figure 6:**
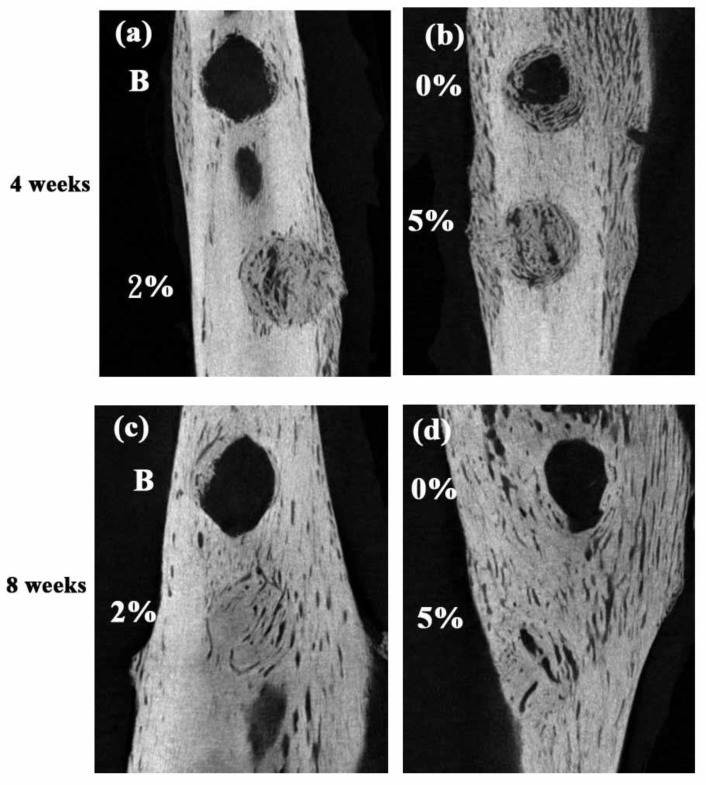
Micro-CT images of artificial defects after four weeks (**a**,**b**) and eight weeks (**c**,**d**). (**a**,**c**) show blank control (upper defects) and 2% Fe_3_O_4_/PLLA nanofiber-filled samples (lower defects), respectively; (**b**,**d**) show neat PLLA (upper defects) and 5% Fe_3_O_4_/PLLA (lower defects) nanofiber-filled samples, respectively.

Figure 6 shows micro-CT images of the four samples obtained after four weeks (a and b) and eight weeks (c and d). The images confirm the absence of newly-formed bone in the blank control sample after four weeks. However, new bone formation is observed in the samples grafted with neat PLLA and Fe_3_O_4_-PLLA nanofibers. For the control fill-free samples, mineralization tissue is observed in the marginal region of the defect only after a healing time of eight weeks (Figure 6c). However, loose newly-formed bone can be seen in the marginal area of the defect filled with neat PLLA nanofibers after four weeks (Figure 6b). For the neat PLLA-filled sample, the loose newly formed bone transforms to a dense analogous material after eight weeks (Figure 6d). For the samples grafted with nanofibers containing Fe_3_O_4_ nanoparticles, newly-formed bone extends to the central area of the grafting site after four weeks (Figure 6a,b). Furthermore, after eight weeks of healing, no visible margin is discerned, irrespective of the Fe_3_O_4_ nanoparticle concentration (2% or 5%).

**Figure 7:**
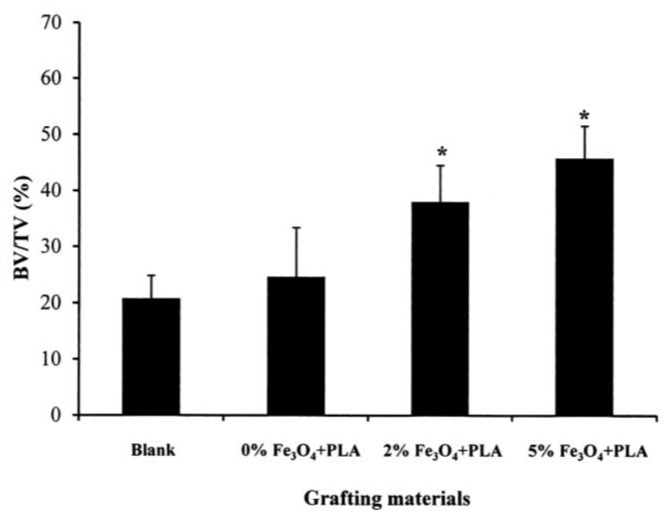
BV/TV% values for artificial bony defects filled with blank, neat PLLA and Fe_3_O_4_/PLLA nanofibers after eight weeks (* *p* < 0.05).

Figure 7 presents the quantitative results obtained from the micro-CT images for the bone volume-to-tissue volume (BV/TV) ratios of the four samples after an eight-week healing time. No significant difference is observed between the blank control sample and the sample filled with neat PLLA nanofibers. However, for the bone defects grafted with Fe_3_O_4_-PLLA nanofibers, the BV/TV ratios are significantly increased. From inspection, the BV/TV ratios of the samples grafted with 2% and 5% Fe_3_O_4_-PLLA nanofibers are equal to 38.05 ± 6.46% and 45.79 ± 5.69%, respectively, and are hence 1.9 and 2.3 times higher than that of the blank sample (20.81 ± 4.02%).

## 4. Discussion

PLLA has long been used to fabricate biodegradable materials since its debris can be naturally metabolized to become carbon dioxide and water [36]. However, when PLLA is mixed with other materials, the biocompatibility of these additives becomes an important concern. Previous studies have shown that Fe_3_O_4_ nanoparticles have an osteogenic effect on bone cells [26,28,37]. Chang et al. (2015) mixed Fe_3_O_4_ nanoparticles into PLLA to form a biodegradable and radiopaque material for X-ray imaging-enhanced bone screws. After implanting these screws in rabbit tibias, they found that nano-Fe_3_O_4_/PLLA screws accelerated the bone healing process [38]. The CT images showed that the Fe_3_O_4_ nanoparticle/PLLA screws exhibited a 1.5-fold higher bone volume growth than that of neat PLLA screws after eight weeks when implanted in rabbit tibias. The results presented in Figure 7 of the present study similarly indicate that bony defects grafted with Fe_3_O_4_/PLLA nanofibers demonstrate a higher bone healing activity. These results confirm the findings of Chang et al. (2015).

The literature contains many studies on the incorporation of Fe_3_O_4_ nanoparticles into nanofibers using an electrospinning technique [28,29,31,37,39,40]. However, most of these studies focus on the physical properties of the nanofibers or the development of novel fabrication techniques. While Wei et al. [39] and Wang et al. [29] provided evidence to show the bone regenerative effect of these novel materials, they only conducted in vitro cellular experiments. By contrast, the present study has employed animal study and, for the first time, has presented in vivo evidence to confirm the effectiveness of Fe_3_O_4_/PLLA nanofibers in promoting bone growth.

The histological results presented in this study have shown evidence of loose fibrous connective tissue, inflammatory cells, and blood clotting in the implantation site one week after grafting (Figure 4a). This finding is consistent with that of a previous report on dental implant healing [41], which showed an abundance of clotted blood and a severe inflammation response in the early stage of bone healing. It is well known that interactions between inflammatory cells and cells related to bone healing play a key role in the repair and remodeling of bone [42,43,44]. Since inflammatory cells are the primary source of inflammatory signals during the initial stage of bone healing, the acute inflammation observed in the present study after a healing time of two weeks (Figure 4b) can be recognized as the first stage of the bone healing process [45]. As shown in Figure 4c, newly formed bone existed at the margin of the grafting site four weeks after implantation. This phenomenon is consistent with the standard bone healing process, in which bone formation begins in the inner layer of the periosteum at some distance from the injury site [46,47,48]. Furthermore, the appearance of woven bone represents the first stage in the mineralization of fibrous tissue [42]. After eight weeks of healing, osteoblast and osteoclast are both present in the grafting site (Figure 4d). It is well known that the interactions and cross-talk among osteoclast and mesenchymal stem cell (MSC) derived osteoblast are an important mechanism for modulating bone repair. Notably, marrow spaces have also been observed in the grafting site after four weeks. Bone marrow is a known source of osteoprogenitor cells, including MSC. During the process of bone formation and fracture healing, the differentiation of MSC to osteoblasts is a key procedure for bone matrix formation and mineralization [49]. In other words, the presence of marrow spaces in the present sample indicates an on-going process of active bone remodeling in the grafting site.

The implantation sites filled with 2% and 5% Fe_3_O_4_/PLLA nanofibers exhibited significantly higher BV/TV values than the unfilled control sample or neat-PLLA sample after eight weeks of healing (Figure 7). Thus, it seems reasonable to infer that Fe_3_O_4_/PLLA nanofibers have a positive effect on bone healing. The CT images revealed no obvious differences between the drilled holes filled with 2% (Figure 6c) and 5% (Figure 6d) Fe_3_O_4_/PLLA nanofibers, respectively, after eight weeks. The quantitated CT results for the corresponding BV/TV ratios also showed no significant difference (Figure 7). The histological images showed that the major bone tissue after eight weeks in the site grafted with 2% Fe_3_O_4_/PLLA nanofiber was woven bone (Figure 5c). Woven bone is a mineralized tissue characterized by a higher osteocyte density than lamellar bone [50]. It is produced following injury, but is soon replaced with more resilient lamellar bone. Consequently, the present finding of no significant difference between the BV/TV values of the 2% and 5% Fe_3_O_4_/PLLA samples, respectively, may simply reflect the inability of the CT images to distinguish between woven bone and matured lamella bone due to resolution issues. Overall, however, the histological images presented in Figure 5 suggest that the positive effect of Fe_3_O_4_/PLLA nanofibers on bone regeneration is Fe_3_O_4_ nanoparticle dose-dependent. In particular, the bone regeneration effect is enhanced as the dosage of Fe_3_O_4_ nanoparticles increases. This data suggests that the Fe_3_O_4_/PLLA nanofibers used in this study can be a useful biomaterial for bone tissue engineering.

## 5. Conclusions

The Fe_3_O_4_/PLLA nanofibers fabricated in this study have demonstrated a positive effect on the bone healing process in an animal model. As such, they appear to have a promising potential for the grafting of bony defects and treatment of bone fractures. The present results may also serve as a useful reference for future advanced studies on similar nano-composites.

## Figures and Tables

**Table 1:** Quantification of the new bone formation ratio

Samples	Blank	Neat PLLA	2% Fe_3_O_4_/PLLA	5% Fe_3_O_4_/PLLA
	0%	13%	43%	44%

## Supplementary Materials

The following are available online at www.mdpi.com/2073-4360/10/7/804/.
