# Supplementary material for: In Vivo Investigation into Effectiveness of Fe3O4/PLLA Nanofibers for Bone Tissue Engineering Applications
Source: Polymers (Basel). 2018 Jul 22;10(7):804. doi: 10.3390/polym10070804 (PMC6404065; doi:10.3390/polym10070804)
Supplement: Supplementary file 1 [file polymers-10-00804-s001.pdf]

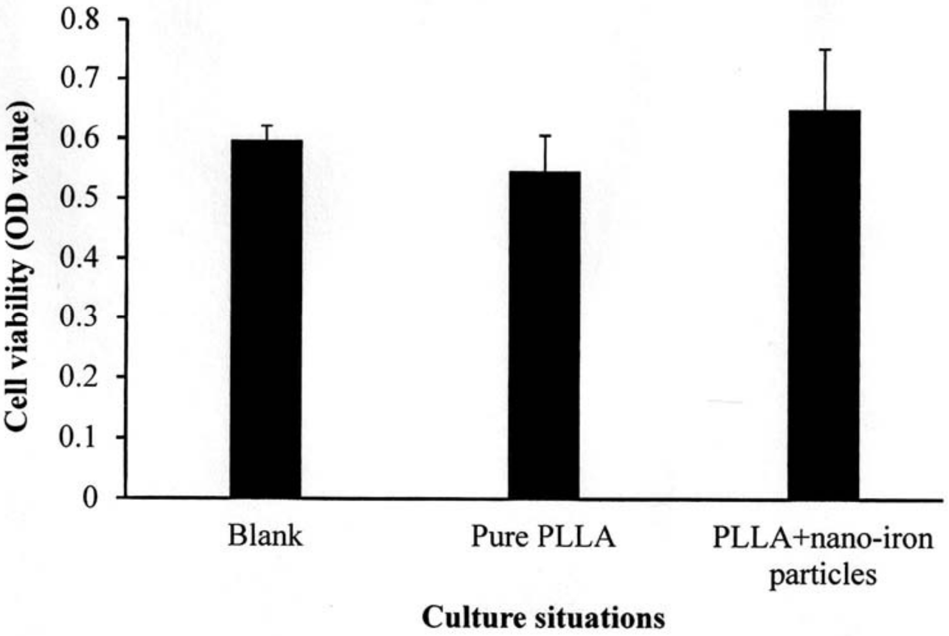

Figure S1. A set of experiment was performed to prove the non- cytotoxic evidence of the nanoparticles/PLLA composite. The cytotoxicity of the nano-Fe_3O_4/PLLA composites was evaluated by determining the viability of MG-63 cells after incubation in medium containing liquid extracts of the composites for 24 h.
